# Clinical Utility of Preoperative Vitamin D3 Injection for Preventing Transient Hypocalcemia after Total Thyroidectomy

**DOI:** 10.1155/2021/6683089

**Published:** 2021-02-13

**Authors:** Kwangsoon Kim, Cho Rok Lee, Sang-Wook Kang, Jandee Lee, Jong Ju Jeong, Kee-Hyun Nam, Woong Youn Chung

**Affiliations:** ^1^Department of Surgery, College of Medicine, The Catholic University of Korea, Seoul, Republic of Korea; ^2^Department of Surgery, Yonsei University College of Medicine, Seoul, Republic of Korea

## Abstract

**Background:**

Postoperative transient hypocalcemia (TH) is a common complication of total thyroidectomy. This retrospective study evaluated the clinical utility of preoperative vitamin D3 injection for the prevention of TH after total thyroidectomy.

**Methods:**

We included 2294 patients who underwent total thyroidectomy from January 2015 until October 2018 and retrospectively analyzed their data by complete chart review at our hospital. The patients were divided into two groups: vitamin D3 injection (VDI; *n* = 342) and vitamin D3 noninjection (VDN; *n* = 1952). TH was defined as serum calcium <8.2 mg/dL and signs or symptoms of hypocalcemia.

**Results:**

The mean preoperative serum 25-hydroxyvitamin D (25-OHD) levels of the VDI group were significantly lower than those of the VDN group (16.5 ± 6.9 ng/mL vs 19.4 ± 8.7 ng/mL, *p* < 0.001). Multivariate analysis indicated that the significant risk factors of TH include vitamin D noninjection (hazard ratio (HR): 1.717, 95% confidence interval (CI): 1.282–2.300, *p* < 0.001), male gender (HR: 1.427, 95% CI: 1.117–1.822, *p* = 0.004), and capsular extension (HR: 1.273, 95% CI: 1.011–1.603, *p* = 0.040).

**Conclusions:**

Preoperative vitamin D3 injection significantly contributed to the prevention of TH after total thyroidectomy. Further prospective or multicenter studies must be conducted to determine the effect of vitamin D3 injection.

## 1. Introduction

Total thyroidectomy (TT) has been accepted as the treatment of choice for some malignant and benign thyroid diseases [1]. However, TT can cause complications; two common complications associated with TT are damage to the recurrent laryngeal nerve and damage to parathyroid glands due to close anatomic proximity to the thyroid gland [2–4].

The most frequent complication following TT is transient hypocalcemia (TH), which accounts for 2% to 83% of all complications depending on the definition [5–7]. TH, secondary to hypoparathyroidism, occurs due to intraoperative injury to the parathyroid glands or their blood supply, or unintentional removal of the parathyroid glands [8]. Factors that increase the risk of hypocalcemia include the difficulty of operation, such as in advanced thyroid cancer, Graves' disease, huge goiters, history of previous neck operation, and metastasis to lymph nodes in the central compartment [9–11]. Several unpleasant symptoms of hypocalcemia, such as paresthesia, spasm, tetany, tingling sensation, and even arrhythmia in severe cases, result in an increase in the length of hospital stay and overall treatment cost [11]. Post-TT hypoparathyroidism induces a decrease in circulating levels of parathyroid hormone (PTH). Low levels of PTH reduce renal calcium reabsorption and calcium release from bones and, subsequently, serum calcium levels are decreased.

Vitamin D plays an essential role in calcium metabolism by increasing calcium absorption from the gastrointestinal tract [12]. Vitamin D deficiency (VDD), defined as serum 25-hydroxyvitamin D (25-OHD) level of less than 10 ng/mL, is common in South Korea and has a prevalence of 47.3% in males and 64.5% in females [13]. As a compensatory mechanism of VDD to maintain serum calcium levels, VDD leads to increased PTH secretion from parathyroid glands and thereby improves calcium absorption by enhancing renal calcium reabsorption. Some studies have demonstrated that VDD is an independent risk factor for postoperative hypocalcemia after TT [14, 15].

The specific treatment of TH is oral vitamin D (calcitriol) and calcium supplements, which can alleviate symptoms and normalize serum calcium levels. Some authors have suggested that oral vitamin D and calcium supplements should be routinely administered prophylactically during the postoperative period to reduce the incidence of symptomatic hypocalcemia [16, 17]. However, there have been no studies on the efficacy of preoperative vitamin D3 injection for the prevention of TH after TT.

This retrospective study was conducted to evaluate the clinical utility of preoperative vitamin D3 injection for the prevention of TH.

## 2. Materials and Methods

### 2.1. Patients

We retrospectively reviewed the medical records of 2457 patients who underwent TT at Yonsei University Hospital (Seoul, Korea) between January 2015 and October 2018; 102 patients were excluded based on a diagnosis of permanent hypocalcemia, and 61 patients were excluded as they were lost to follow-up or had inadequate follow-up data. The final analysis dataset included 2294 patients whose data were collected and analyzed based on a complete review of medical charts and pathology reports. This study was approved by Yonsei University institutional review board (IRB no.:4-2020-0039), which waived the requirement for informed consent due to the retrospective nature of this study.

### 2.2. Definitions of Study Variables

According to World Health Organization, serum 25-OHD levels ≥30, 10 to 29, and <10 ng/mL are defined as sufficiency, insufficiency, and deficiency, respectively [18]. Postoperative hypocalcemia was defined as serum calcium concentration <8.2 mg/dL without or with the presence of any symptoms or signs, such as numbness, paresthesia, Trousseau's sign, Chvostek's sign, or tetany. The normal level of intact PTH (iPTH) was 15 to 65 pg/mL and was measured preoperatively, 1 hour after TT, and at 6 AM on the first postoperative day. The normal levels of serum calcium, phosphorous (P), and ionized calcium were 8.2 to 10.2, 2.5 to 4.2, and 4.5 to 5.2 mg/dL, respectively, at the Yonsei University Hospital. Each level was measured preoperatively, 1 hour after TT, and at 6 AM every day until discharge.

### 2.3. Protocol for Vitamin D3 Injection

Serum 25-OHD levels were preoperatively measured within 1 month of TT in all patients who planned to undergo TT. The decision to administer a vitamin D3 injection was determined by the patient's serum 25-OHD levels, age, sex, and planned surgical extent. Patients with 25-OHD level below 30 ng/mL were primary candidates for vitamin D3 injections. However, some patients with 25-OHD level above 30 ng/mL also received injections when the following medical conditions were present: advanced thyroid cancer and underlying diseases such as diabetes mellitus, chronic kidney disease, and osteoporosis. The injection was administered within 1 week before the operation in an outpatient setting. In total, 342 patients were injected with 200,000 IU of Vitamin D3 B.O.N (Haupt Pharma Livron S.A.S, Libron-sur-Drome, France), administered as an intramuscular injection into the buttocks.

### 2.4. Postoperative Management

The policy for management of TH at our institution includes oral calcium (3–6 g/day) and/or oral 1, 25-dihydroxy vitamin D3 (calcitriol 1–1.5 *µ*g/day) to all patients with biochemical hypocalcemia. Symptomatic hypocalcemia was treated with parenteral calcium and oral calcitriol. Patients with biochemical hypocalcemia were discharged on oral calcium when calcium levels were in the normal range. Patients with symptomatic hypocalcemia were discharged on oral calcium and/or calcitriol at doses that varied by the calcium level of each patient.

### 2.5. Statistical Analysis

All statistical analyses were conducted in the SPSS software package (SPSS, version 24.0 for Windows; SPSS, Chicago, IL). Continuous variables were reported as the mean with the standard deviation (SD) and categorical variables were reported as the numbers with the percentage. Student's *t*-test, chi-square test, or Fisher's exact test, if necessary, had been used to compare two groups. Multivariate logistic regression analyses were carried out to validate which factors were associated with TH. Odds ratios (ORs) with 95% confidence intervals (CI) were calculated to compare the risk of TH between the independent factors by using linear logistic regression analysis. A statistically significant difference was defined as *p* < 0.05.

## 3. Results

The mean age of the study population was 45.5 ± 13.6 years (range, 8–84 years) and 1,746 (76.1%) patients were female. The majority of patients (*n* = 2256, 98.3%) were diagnosed with papillary thyroid carcinoma (PTC). Patients were divided into two groups depending on whether they received preoperative vitamin D3 injections: 342 (14.9%) patients received vitamin D3 injection, and 1952 (85.1%) patients did not receive the injection.

### 3.1. Comparison of Baseline Clinicopathologic and Perioperative Biochemical Characteristics between Vitamin D3 Injection (VDI) and Vitamin D3 Noninjection (VDN) Groups

Table 1 presents a comparison of baseline clinicopathologic characteristics between the VDI and the VDN groups. There were no statistically significant intergroup differences in mean age, proportion of female patients, multifocality, bilaterality, extrathyroidal extension (ETE), mean tumor size, and TNM staging. However, the incidence of TH was significantly lower in the VDI group than in the VDN group (19.6% vs 29.3%, *p* < 0.001).

**Figure 1:**
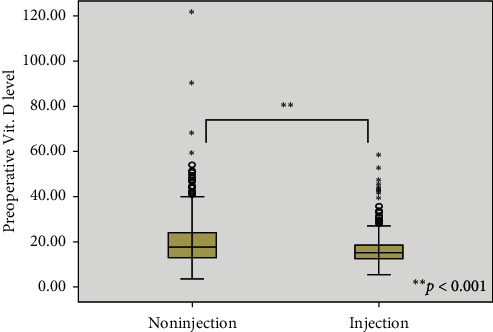
Comparison of preoperative 25-hydroxyvitamin D (25-OHD) levels between vitamin D3 injection (VDI) and vitamin D3 noninjection (VDN) groups.

The results of comparison of the perioperative biochemical characteristics between the VDI and the VDN groups are shown in Table 2. Perioperative biochemical factors, preoperative 25-OHD, PTH, Ca, P, and ionized Ca were compared between the two groups but did not differ significantly except for preoperative 25-OHD level, which was significantly lower in the VDI group than in the VDN group (16.5 ± 6.9 ng/mL vs 19.4 ± 8.7 ng/mL, *p* < 0.001, Figure 1).

### 3.2. Comparison of Baseline Clinicopathologic and Perioperative Biochemical Characteristics of Patients with TH between VDI and VDN Groups

A total of 638 patients were postoperatively diagnosed with TH (VDI group *n* = 67 (10.5%); VDN group *n* = 571 (89.5%)). There were no significant intergroup differences except for the mean operative time and M stage (Table 3). The mean operation time for the VDI group was significantly longer than that of the VDN group (176.7 ± 210.3 vs 150.7 ± 70.4 minutes, *p* = 0.034). On the basis of M stages, the VDI group had a significantly higher tumor grade (*p* = 0.028).

Table 4 presents the comparison of perioperative biochemical characteristics in patients with TH between the VDI and the VDN groups. Similar to Table 2, there was no significant difference between the two groups except for the preoperative 25-OHD level. The preoperative 25-OHD level was significantly lower in the VDI group than in the VDN group (15.2 ± 6.0 ng/mL vs 19.2 ± 8.4 ng/mL, *p* < 0.001).

### 3.3. Multivariate Analysis of Clinical Parameters Which Influence TH

Multivariate logistic regression analyses were conducted to identify the independent risk factors associated with TH (Table 5). Male gender (OR, 1.427; 95% CI, 1.117–1.822; *p* = 0.004) and ETE (OR, 1.273; 95% CI, 1.011–1.603; *p* = 0.040) were confirmed as significant predictors for TH. Among the various risk factors, injection of vitamin D3 was identified as the most significant predictor for TH (OR, 1.717; 95% CI, 1.282–2.300; *p* < 0.001).

### 3.4. Subgroup Analysis between VDI and VDN Groups according to Vitamin D Level Range and in Postoperative PTH Range from 10 to 15

**Figure 2:**
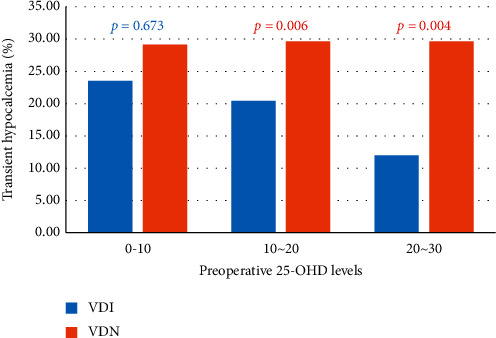
Comparison of incidence of postoperative transient hypocalcemia between vitamin D3 injection (VDI) and vitamin D3 noninjection (VDN) groups according to preoperative 25-hydroxyvitamin D (25-OHD) levels.

We undertook subgroup analysis to identify the differences in incidence of TH according to preoperative 25-OHD levels between the two groups. The patients were divided into the following three levels: low level (0–10 ng/mL, *n* = 185, 8.1%), intermediate level (10–20 ng/mL, *n* = 1160, 50.6%), and high level (20–30 ng/mL, *n* = 735, 32.0%). In the low 25-OHD level, there was no statistically significant difference in the incidence of TH between the VDI and VDN groups (23.5% vs 29.1%, *p* = 0.673). However, the incidence of TH was significantly lower in the VDI group than in the VDN group in both intermediate and high 25-OHD levels (20.4% vs 29.6%, *p* = 0.006 in intermediate level; 11.9% vs 27.9%, *p* = 0.004 in high level; Table 6 and Figure 2).

A total of 250 (10.9%) patients had postoperative PTH levels of 10 to 15 pg/mL.  Table 7 presents a subgroup analysis between the VDI and VDN groups of the postoperative PTH range from 10 to 15 pg/mL. There was no statistically significant difference in the incidence of TH between the two groups, although PTH incidence was relatively lower in the VDI group (30.6% vs 46.7%, *p* = 0.051).

### 3.5. Linear Logistic Regression Analysis between TH and Independent Variables

Table 8 shows the results of linear logistic regression analysis on which independent risk factors are associated with TH. The combination of intermediate level of 25-OHD and noninjection of vitamin D3 was significantly associated with TH (OR, 1.603; 95% CI, 1.001–2.567; *p* = 0.050). Moreover, the combination of low level of 25-OHD and noninjection of vitamin D3 was more likely to be associated with TH (OR, 1.662; 95% CI, 1.178–2.345; *p* = 0.004).

## 4. Discussion

TH is a common complication after TT. The incidence of this complication accounts for 2 to 83% depending on the definition of TH [5–7]. In general, TH is defined as a clinicopathological state necessitating exogenous calcium supplements either to maintain normal serum calcium levels or to correct clinical signs and symptoms of hypocalcemia [19]. Although most patients recover from TH, permanent hypocalcemia can occur in 0 to 13% of patients who undergo TT [20]. In particular, permanent hypocalcemia significantly decreases the quality of life of patients. Therefore, it is crucially important for the surgeon to prevent postoperative hypoparathyroidism [21, 22]. Various clinical, biological, and surgical factors may influence the development of TH after TT. The main factor among these various factors would be intraoperative trauma to the parathyroid glands or their blood supply, or unintentional intraoperative removal of parathyroid glands, which is anatomically close to the thyroid gland [8]. However, it would be controversial to attribute all causes of TH after TT to surgical factors. Many clinicopathologic factors are involved in TH, such as age, extent of operation, thyroid diseases, neck dissection, and preoperative biochemical status.

In particular, vitamin D plays an important role in calcium homeostasis [12]. Vitamin D is referred to as D2 or D3 or both together. Vitamin D3 is produced by exposure to sunlight on the skin. Hydroxylation occurs in the liver to form 25-OHD, and 25-OHD is hydroxylated in the kidney to form 1, 25-hydroxyvitamin D. The hydroxylation in the kidney is the major control point in vitamin D metabolism. Factors affecting hydroxylation are phosphate, serum PTH, and calcium concentrations. Reduced PTH and phosphate concentrations stimulate independently to increase renal production of 1, 25-hydroxyvitamin D, which increases bone resorption, enhances the effects of PTH in the kidney to facilitate renal calcium reabsorption, and increases absorption of calcium from the gut [23].

Several studies have reported a role of preoperative serum 25-OHD, as to whether VDD contributes to TH. Lin et al. reported that VDD did not increase the rate of hypocalcemia after near-total thyroidectomy [24]. However, all patients in this study were routinely supplemented with oral calcium carbonate, 500 mg 3 times daily, and vitamin D (cholecalciferol), 2000 IU daily. Cherian et al. reported that VDD did not increase the risk of postoperative hypocalcemia as well [25]. On the other hand, Yamashita et al. reported association between VDD and TH [15]. However, their study was conducted among patients with Graves' disease. Danan et al. reported that VDD was a significant risk predictor of postoperative hypocalcemia in a series of 67 patients undergoing TT [26]. In addition, Erbil et al. reported that patients with advanced age and low preoperative serum 25-OHD levels should be supplemented with oral calcium or vitamin D after TT to avoid TH [14]. We hypothesized that raising the 25-OHD levels before the operation may prevent postoperative hypocalcemia.

We investigated risk factors associated with TH after TT in 2294 patients with thyroid cancer. The incidence of TH in the present study was 27.8%, which was not significantly different from previous studies [27–29]. The mean preoperative 25-OHD level of study population was 18.9 ng/mL, which was insufficient state (>30 ng/mL, sufficiency). The preoperative 25-OHD level was significantly lower in the VDI group than in the VDN group (16.5 ng/mL vs 19.4 ng/mL, *p* < 0.001, Figure 1). Interestingly, there was a statistically significant difference in the incidence of TH between the VDI and VDN groups (19.6% vs 29.3%, *p* < 0.001). These results cannot simply be interpreted to mean that vitamin D3 injection prevented TH. Serum 25-OHD levels are slightly increased 4 weeks after injection of vitamin D3 [30, 31]. The patients in the present study were administered a single intramuscular injection of vitamin D3 (200,000 IU) within 1 week before the operation, but no blood test was done to check by how much the 25-OHD levels were elevated after the vitamin D3 injection. Nevertheless, VDI was identified as the most significant predictors for TH on multivariate analysis (OR, 1.717; 95% CI, 1.282–2.300; *p* < 0.001). Our institution is a leading tertiary center in thyroid surgery and most patients are referred from other hospitals with known diagnosis of thyroid cancer. Since preoperative study and operation are the routine course for most patients, serum 25-OHD level after vitamin D3 injection could not be evaluated. A prospective study on this subject will be performed in the future in order to determine the correlation between vitamin D3 injection and serum 25-OHD level.

We confirmed the effect of vitamin D3 injection according to the 25-OHD levels. In patients with low levels of 25-OHD (0–10 ng/mL), vitamin D3 injection did not affect the development of TH, whereas in cases with greater than 10 ng/mL 25-OHD levels, the incidence of TH was significantly lower in the VDI group than in the VDN group. Moreover, linear logistic regression analysis showed that the combination of preoperative low 25-OHD and no injection of vitamin D3 was more likely to be associated with TH.

Vitamin D and PTH interact individually and synergistically to maintain calcium homeostasis [32]. Low levels of PTH are potent stimulants of the hydroxylation of 25-OHD, and reduced levels of 25-OHD stimulate PTH secretion. Therefore, we conducted a subgroup analysis of postoperative PTH levels from 10 to 15 pg/mL to confirm the differences of TH between the VDI and VND groups. There was no statistically significant intergroup difference. However, the incidence of TH was relatively lower in the VDI group than in the VDN group (30.6% vs 46.7%, *p* = 0.051). This means that vitamin D3 injection might prevent TH, especially in patients with postoperatively borderline-low PTH levels. Jung et al. reported that decline of postoperative PTH levels can be used as a predictive factor for TH [33]. Vitamin D3 injection may be helpful in situations where PTH levels may be decreased postoperatively, such as in thyroiditis, extensive lymph nodes metastasis at level VI, Graves' disease, history of previous neck operation, and extensive surgical resection. Because it is difficult to conserve parathyroid glands or their blood supplies in these situations, PTH levels can be decreased postoperatively. One of the possible mechanisms why vitamin D3 injection may prevent TH in patients with hypoparathyroidism after TT is that increased extrarenal hydroxylation of 25-OHD independent of PTH after vitamin D3 injection may maintain serum 1, 25 hydroxyvitamin D levels. Further studies is necessary to validated this hypothesis.

This present study has some limitations. The first limitation is the retrospective study design. In addition, there is a possibility of selection bias, because all enrolled patients were from a single tertiary-care institution. Secondly, there was no significant difference in the total operation time for all patients included in the study. However, within the group where TH was diagnosed postoperatively, VDI group showed a significantly longer mean operation time compared to VDN group. Although the data was not statistically significant, it may have a correlation with the relatively aggressive features in VDI group compared to VDN group. The most important limitation is the timing of the vitamin D3 injection. As mentioned earlier, serum 25-OHD levels are slightly increased 4 weeks after a vitamin D3 injection. However, the study participants received vitamin D3 injection within 1 week before operation, and we did not recheck the 25-OHD levels after the injection. Moreover, serum 1, 25 hydroxyvitamin D levels were not measured. In our institution, the assessment of patients' vitamin D levels has been performed with only measurement of 25-OHD. Finally, we performed vitamin D3 injection after considering clinical features such as preoperative 25-OHD level, age, extent of operation, and comorbidity. However, no definitive indications for vitamin D3 injection before thyroid surgery have been firmly established.

The most important strength of this study is that every patient was followed up using a standardized protocol in a single institution, including the preoperative evaluation of 25-OHD levels and vitamin D3 injection. Moreover, this study included a large cohort, including only patients undergoing TT, and did not include any cases of subtotal or near-total thyroidectomy.

## 5. Conclusions

TH is one of the major postoperative complications of TT. Preoperative vitamin D3 injection has a significant effect toward the prevention of TH after TT. Further prospective or multicenter studies must be conducted to determine the effect of vitamin D3 injection in the clinical setting of TT.

## Figures and Tables

**Table 1:** Comparison of baseline clinicopathologic characteristics between vitamin D3 injection (VDI) and vitamin D3 noninjection (VDN) groups.

	VDI (*n* = 342)	VDN (*n* = 1952)	*p* value
Age (years)	44.3 ± 13.0 (range, 12–81)	45.8 ± 13.7 (range, 8–84)	0.051
Female	253 (74.0%)	1493 (76.5%)	0.336
Multifocality	193 (56.4%)	1114 (57.1%)	0.859
Bilaterality	167 (48.8%)	894 (45.8%)	0.410
ETE	61 (17.8%)	308 (15.8%)	0.339
Operation time (min)	149.8 ± 78.7 (range, 48–835)	150.1 ± 75.6 (range, 50–635)	0.946
Hospital stay (day)	4.0 ± 1.8 (range, 2–17)	3.9 ± 1.7 (range, 2–37)	0.074
Tumor size (cm)	1.4 ± 1.0 (range, 0.2–6.3)	1.3 ± 0.9 (range, 0.2–10.5)	0.259

T stage			0.184
T1	249 (72.8%)	1466 (75.1%)	
T2	32 (9.4%)	169 (8.7%)	
T3	9 (2.6%)	97 (5.0%)	
T4a	52 (15.2%)	213 (10.9%)	
T4b	0 (0%)	4 (0.2%)	

N stage			0.082
N0	113 (33.0%)	667 (34.2%)	
N1a	101 (29.5%)	693 (35.5%)	
N1b	128 (37.5%)	592 (30.3%)	
M stage			0.156
M1	6 (1.8%)	18 (0.9%)	

TNM stage			0.530
Stage I	287 (83.9%)	1597 (81.8%)	
Stage II	39 (11.4%)	278 (14.2%)	
Stage III	15 (4.4%)	65 (3.3%)	
Stage IVa	0 (0%)	2 (0.1%)	
Stage IVb	1 (0.3%)	7 (0.4%)	

Transient hypocalcemia	67 (19.6%)	571 (29.3%)	<0.001

Data are expressed as patient's number (%), or mean ± SD. A statistically significant difference was defined as *p* < 0.05. ETE, extrathyroidal extension; T, tumor; N, node; M, metastasis.

**Table 2:** Comparison of perioperative biochemical characteristics between vitamin D3 injection (VDI) and vitamin D3 noninjection (VDN) groups.

	VDI (*n* = 342)	VDN (*n* = 1952)	*p* value
Preop. 25-OHD (ng/mL)	16.5 ± 6.9 (range, 5.5–55.5)	19.4 ± 8.7 (range, 3.5–87.4)	<0.001
Preop. PTH (pg/mL)	45.9 ± 15.4 (range, 5.8–119.8)	44.7 ± 16.5 (range, 4.7–176.2)	0.197
Postop. PTH (pg/mL)	21.6 ± 14.4 (range, 1.8–90.9)	22.2 ± 16.0 (range, 1.2–126.7)	0.469
Preop. Ca (mg/dL)	9.6 ± 0.4 (range, 6.1–11.1)	9.6 ± 0.4 (range, 7.7–11.9)	0.578
Postop. Ca (mg.dL)	8.1 ± 0.6 (range, 6.4–9.7)	8.2 ± 0.6 (range, 6.0–10.7)	0.373
Preop. P (mg/dL)	3.7 ± 0.5 (range, 2.1–5.4)	3.6 ± 0.5 (range, 2.0–5.7)	0.272
Postop. P (mg/dL)	4.3 ± 0.8 (range, 2.1–7.3)	4.3 ± 0.7 (range, 2.0–7.9)	0.928
Preop. Ionized Ca (mg/dL)	4.8 ± 0.2 (range, 4.0–5.4)	4.8 ± 0.2 (range, 3.1–6.2)	0.286
Postop. Ionized Ca (mg/dL)	4.4 ± 0.2 (range, 3.6–5.2)	4.4 ± 0.3 (range, 3.3–5.8)	0.139

Data are expressed as mean ± SD. A statistically significant difference was defined as *p* < 0.05. preop., preoperative; postop., postoperative; 25-OHD, 25-hydroxyvitamin D; PTH, parathyroid hormone; Ca, calcium; P, phosphorus.

**Table 3:** Comparison of baseline clinicopathologic characteristics of patients with transient hypocalcemia between vitamin D3 injection (VDI) and vitamin D3 noninjection (VDN) groups.

	VDI (*n* = 67)	VDN (*n* = 571)	*p* value
Age (years)	46.1 ± 14.1 (range, 16–78)	45.0 ± 13.7 (range, 8–84)	0.538
Female	54 (80.6%)	460 (80.6%)	0.967
Multifocality	42 (62.7%)	331 (58.0%)	0.513
Bilaterality	31 (46.3%)	266 (46.6%)	0.961
ETE	18 (26.9%)	97 (17.0%)	0.063
Operation time (min)	176.7 ± 210.3 (range, 48–490)	150.7 ± 70.4 (range, 50–635)	0.034
Hospital stay (day)	4.1 ± 1.2 (range, 3–7)	3.9 ± 1.5 (range, 2–23)	0.315
Tumor size (cm)	1.4 ± 1.2 (range, 0.2–6.3)	1.3 ± 0.9 (range, 0.2–10.5)	0.404

T stage			0.135
T1	43 (64.2%)	424 (74.3%)	
T2	6 (8.9%)	46 (8.0%)	
T3	3 (4.5%)	34 (6.0%)	
T4a	15 (22.4%)	65 (11.4%)	
T4b	0 (0%)	1 (0.2%)	

N stage			0.129
N0	23 (34.3%)	192 (33.6%)	
N1a	16 (23.9%)	199 (34.9%)	
N1b	28 (41.8%)	180 (31.5%)	

M stage			0.028
M1	3 (4.5%)	4 (0.7%)	

TNM stage			0.608
Stage I	51 (76.1%)	475 (83.2%)	
Stage II	13 (19.4%)	78 (16.7%)	
Stage III	3 (4.5%)	15 (2.6%)	
Stage IVa	0 (0%)	1 (0.2%)	
Stage IVb	0 (0%)	1 (0.2%)	

Data are expressed as patient's number (%), or mean ± SD. A statistically significant difference was defined as *p* < 0.05. ETE, extrathyroidal extension; T, tumor; N, node; M, metastasis.

**Table 4:** Comparison of perioperative biochemical characteristics of patients with transient hypocalcemia between vitamin D3 injection (VDI) and vitamin D3 noninjection (VDN) groups.

	VDI (*n* = 67)	VDN (*n* = 571)	*p* value
Preop. 25-OHD (ng/mL)	15.2 ± 6.0 (range, 6.3–41.5)	19.2 ± 8.4 (range, 4.2–56.4)	<0.001
Preop. PTH (pg/mL)	47.7 ± 2.2 (range, 22.5–94.1)	45.3 ± 17.5 (range, 19.2–166.2)	0.276
Postop. PTH (pg/mL)	8.1 ± 14.4 (range, 3.8–15.6)	8.0 ± 5.9 (range, 1.2–90.2)	0.982
Preop. Ca (mg/dL)	9.5 ± 0.3 (range, 8.7–10.4)	9.6 ± 0.4 (range, 7.7–10.7)	0.370
Postop. Ca (mg.dL)	7.7 ± 0.5 (range, 6.4–9.0)	7.8 ± 0.5 (range, 6.0–9.1)	0.868
Preop. P (mg/dL)	3.7 ± 0.5 (range, 2.6–4.9)	3.7 ± 0.5 (range, 2.3–5.7)	0.280
Postop. P (mg/dL)	4.8 ± 0.9 (range, 2.8–7.3)	4.7 ± 0.8 (range, 2.0–7.9)	0.156
Preop. Ionized Ca (mg/dL)	4.8 ± 0.2 (range, 4.4–5.1)	4.8 ± 0.2 (range, 4.3–5.6)	0.368
Postop. Ionized Ca (mg/dL)	4.2 ± 0.2 (range, 3.9–4.6)	4.2 ± 0.2 (range, 3.5–4.8)	0.637

Data are expressed as mean ± SD. A statistically significant difference was defined as *p* < 0.05. preop., preoperative; postop., postoperative; 25-OHD, 25-hydroxyvitamin D; PTH, parathyroid hormone; Ca, calcium; P, phosphorus.

**Table 5:** Multivariate analysis of clinical parameters which influence transient hypocalcemia.

	OR (95% CI)	*p* value
Vitamin D3		
Injection	Ref.	
Noninjection	1.717 (1.282–2.300)	<0.001

Gender		
Female	Ref.	
Male	1.427 (1.117–1.822)	0.004
ETE	1.273 (1.011–1.603)	0.040

Data are expressed as odds ratio (OR) and 95% confidence interval (CI). A *p* value <0.05 was considered statistically significant. ETE, extrathyroidal extension.

**Table 6:** Subgroup analysis between vitamin D3 injection (VDI) and vitamin D3 noninjection (VDN) groups according to the 25-OHD levels.

25-OHD levels (0∼10 ng/mL)	VDI (*n* = 34)	VDN (*n* = 151)	*p* value
Age (years)	43.8 ± 13.2 (range, 20–65)	45.5 ± 12.5 (range, 16–80)	0.487
Female	26 (76.5%)	141 (93.4%)	0.007
Multifocality	20 (58.8%)	80 (53.0%)	0.573
Bilaterality	14 (41.2%)	62 (41.1%)	0.569
ETE	7 (20.6%)	23 (15.2%)	0.012
Transient hypocalcemia	8 (23.5%)	44 (29.1%)	0.673

25-OHD levels (10∼20 ng/mL)	VDI (*n* = 240)	VDN (*n* = 920)	*p* value
Age (years)	44.0 ± 12.9 (range, 12–78)	44.3 ± 13.1 (range, 14–84)	0.715
Female	175 (72.9%)	722 (78.5%)	0.070
Multifocality	131 (54.6%)	529 (57.5%)	0.422
Bilaterality	100 (41.7%)	427 (46.4%)	0.191
ETE	43 (17.9%)	164 (17.8%)	0.988
Transient hypocalcemia	49 (20.4%)	275 (29.6%)	0.006

25-OHD levels (20∼30 ng/mL)	VDI (*n* = 59)	VDN (*n* = 676)	*p* value
Age (years)	44.8 ± 15.5 (range, 19–81)	49.9 ± 13.8 (range, 12–82)	0.007
Female	47 (79.7%)	503 (74.4%)	0.436
Multifocality	37 (62.7%)	389 (57.5%)	0.493
Bilaterality	30 (50.8%)	310 (45.9%)	0.497
ETE	11 (18.6%)	121 (17.9%)	0.747
Transient hypocalcemia	7 (11.9%)	189 (27.9%)	0.004

Data are expressed as patient's number (%), or mean ± SD. A statistically significant difference was defined as *p* < 0.05. 25-OHD, 25-hydroxyvitamin D; ETE, extrathyroidal extension.

**Table 7:** Subgroup analysis between vitamin D3 injection (VDI) and vitamin D3 noninjection (VDN) groups in postoperative PTH range from 10 to 15 pg/mL.

	VDI (*n* = 36)	VDN (*n* = 214)	*p* value
Age (years)	49.8 ± 13.2 (range, 25–81)	45.9 ± 14.5 (range, 16–82)	0.135
Female	30 (83.3%)	172 (80.4%)	0.821
Multifocality	22 (61.1%)	121 (56.5%)	0.717
Bilaterality	15 (41.7%)	93 (43.5%)	0.858
ETE	7 (19.4%)	38 (17.7%)	0.485
Transient hypocalcemia	11 (30.6%)	100 (46.7%)	0.051

Data are expressed as patient's number (%), or mean ± SD. A statistically significant difference was defined as *p* < 0.05. ETE, extrathyroidal extension.

**Table 8:** Linear logistic regression analysis.

Dependent variable	Independent variables	OR (95% CI)	*p* value
Postoperative transient hypocalcemia	25-OHD ≤ 10 (ng/mL) + injection	1.199 (0.511–2.813)	0.676
	10 < 25-OHD ≤ 20 (ng/mL) + no injection	1.603 (1.001–2.567)	0.050
	25-OHD ≤ 10 (ng/mL) + no injection	1.662 (1.178–2.345)	0.004

Data are expressed as odds ratio (OR) and 95% confidence interval (CI). A *p* value <0.05 was considered statistically significant. 25-OHD, 25-hydroxyvitamin D; ETE, extrathyroidal extension.

## Data Availability

The data that support the findings of this study are available on request from the corresponding author. The data are not publicly available due to privacy or ethical restrictions.
